# Supplementary material for: Study protocol of the PEruvian Registry of ST-segment Elevation Myocardial Infarction II (PERSTEMI-II) study
Source: PLoS One. 2021 Sep 17;16(9):e0257618. doi: 10.1371/journal.pone.0257618 (PMC8448363; doi:10.1371/journal.pone.0257618)
Supplement: S1 Table — (DOCX) [file pone.0257618.s001.docx]

**S1 Table. List of variables to be collected.**

|  | **Variable** | **Type** | **Definition** | **Final expression** |
| --- | --- | --- | --- | --- |
| 1 | Age | Quantitative | Age in years at time of study | In years of age |
| 2 | Sex | Categorical | Genetic sex as listed on the patient's ID card | Man - woman |
| 3 | **Management times** |  |  |  |
| 3.1 | Total ischemia time | Quantitative | Time in minutes from onset of angina symptoms to successful reperfusion (fibrinolytic or PCI route) | In minutes |
| 3.2 | Time of first medical contact | Quantitative | Time in minutes from onset of angina symptoms to first evaluation by a health care provider (includes ECG and diagnosis) | In minutes |
| 4 | **History and risk factors** |  |  |  |
| 4.1 | Hypertension | Categorical | History of hypertension in the anamnesis of the patient's admission. | Yes - No |
| 4.2 | Diabetes Mellitus | Categorical | History of type 1 or 2 diabetes mellitus in the patient's admission history. | Yes - No |
| 4.3 | Dyslipidemia | Categorical | History of cholesterol > 200 or LDL > 130 in the patient's admission history. | Yes - No |
| 4.4 | Current smoker | Categorical | Current cigarette smoking history | Yes - No |
| 4.5 | Chronic coronary artery disease | Categorical | History of stable angina pectoris with or without medication prior to the current infarction episode | Yes - No |
| 4.6 | Prior myocardial infarction | Categorical | History of prior myocardial infarction referred by the patient | Yes - No |
| 4.7 | Prior revascularization | Categorical | History of myocardial revascularization | Yes - No |
| 4.7.1 | Previous PCI | Categorical | History of percutaneous angioplasty with or without stenting > 1 month prior to the event | Yes - No |
| 4.7.2 | Previous revascularization surgery | Categorical | History of aorto-coronary bypass > 1 month before the event | Yes - No |
| 4.8 | Chronic kidney disease | Categorical | History of renal insufficiency or creatinine clearance calculated at admission < 60 ml/min | Yes - No |
| 4.9 | Heart failure | Categorical | History of chronic heart failure with or without optimal medication | Yes - No |
| 5 | Clinical Presentation | Categorical | Symptoms of onset of myocardial infarction as reported by the patient or family member at the time of admission | Angina  Dyspnea  Atypical chest pain Syncope  Cardiac arrest |
| 5.1 | If the presentation was cardiac arrest | Categorical | Site of cardiac arrest | Out-of-hospital In-hospital |
| 6 | Hemodynamic status on admission to the hospital center | Categorical | Defined according to the Killip and Kimpbal scale at the time of admission to the emergency department | KK I: No signs of failure KK II: Cramp in bases  KK III: Pulmonary edema  KK IV: Cardiogenic shock |
| 7 | **Electrocardiography** |  |  |  |
| 7.1 | Rhythm in the ECG at admission | Categorical | Cardiologist-assessed rhythm in the first ECG on admission to the emergency room | Sinus  Atrial fibrillation  AV Block II - III |
| 7.2 | Infarct location | Categorical | Localization of ST-segment elevation in the ECG at admission | Anterior : STE V3 -V4 Anteroseptal: STE V1-V4 Anterolateral: STE V3-V6  Anterior extenso: STE V1- V6  Inferior: STE II, III y aVF Inferoposterolateral: STE II, III, aVF, V5, V6 + InfraST V1 - V2 Lateral: STE I, aVL, V5, V6  Right ventricle: V3R, V4R |
| 8 | **Reperfusion therapy** | Categorical | Did the patient receive any type of reperfusion? (fibrinolysis or PCI, regardless of the final result of these) | Yes - No |
| 8.1 | **Fibrinolytics** | Categorical | Did the patient receive a fibrinolytic? | Yes - No |
| 8.1.1 | Fibrinolysis success | Categorical | ST drop > 50% in leads at 90 minutes post tPA, 60 minutes post TNK | Yes - No |
| 8.2 | **Coronary interventionism** | Categorical | Were coronary angiography and PCI performed? | Yes - No |
| 8.2.1. | Reason for Intervention | Categorical | Express why interventionism is performed. | Primary PCI < 12 hours. Primary PCI 12 to 24 hours  Primary PCI > 24 hours  Rescue PCI Drug-invasive strategy |
| 8.2.2 | Time door-balloon | Quantitative | Expresses the time elapsed from arrival of the PCI-capable patient at the hospital to balloon inflation in the ARI | In minutes |
| 8.2.3 | ARI (artery responsible for the infarction) | Categorical | Defined by the artery presenting thrombus image and correlated to ECG findings | Left coronary artery trunk Anterior descending Anterior descending Anterior circumflex Right coronary artery Venous bridge Arterial bridge Unknown |
| 8.2.4 | Initial TIMI flow of the ARI | Categorical | Defined by the initial flow according to the TIMI scale in the artery responsible for the infarct before passing the angioplasty guidewire | TIMI O TIMI 1 TIMI 2 TIMI 3 |
| 8.2.5 | Final TIMI flow of the ARI | Categorical | Defined by the final flow according to the TIMI scale in the artery responsible for the infarction after stent | TIMI O TIMI 1 TIMI 2 TIMI 3 |
| 8.2.6 | Type of stent placed | Categorical | According to the type of stent placed in the ARI | Medicated Stent Non-medicated Stent No Stent Placed |
| 8.2.7 | Other injuries outside the ARI | Categorical | Were there other coronary lesions >70% outside the artery responsible for the infarction? | Yes - No |
| 8.2.7.1 | PCI of injuries other than ARI | Categorical | Was PTCA with or without stent performed in lesions other than ARI? | Yes - No |
| 8.2.7.2 | Timing of PCI to other injuries | Categorical | When was PCI performed in other lesions? | Intraprocedural primary PCI  Deferred prior to discharge |
| 8.2.7.3 | PCI decision to other injuries | Categorical | What was the reason for interventionism to other lesions? | Routine  Indicated for ischemia in stress tests with imaging. |
| 8.2.7.4 | CABG for other injuries | Categorical | Was revascularization surgery decided due to the presence of other lesions outside the ARI? | Yes - No |
| 8.3 | **Complementary therapy** |  |  |  |
| 8.3.1 | Anticoagulation | Categorical | Did the patient receive anticoagulation as adjunctive therapy? | Yes - No |
| 8.3.2 | Aspirin | Categorical | Did the patient receive aspirin? | Yes - No |
| 8.3.4 | Clopidogrel | Categorical | Did the patient receive clopidogrel? | Yes - No |
| 8.3.5 | Prasugrel | Categorical | Did the patient receive prasugrel? | Yes - No |
| 8.3.6 | Ticagrelor | Categorical | Did the patient receive ticagrelor? | Yes - No |
| 8.3.7 | Bivalirudin | Categorical | Did the patient receive bivalirudin in the hemodynamic room? | Yes - No |
| 8.3.8 | GP IIb/IIIa Inhibitor | Categorical | Did the patient receive a glycoprotein Iib/IIIa inhibitor in the hemodynamic room? | Yes - No |
| 8.3.9 | Thrombus aspiration | Categorical | Was intracoronary thrombus aspiration performed during PCI? | Yes - No |
| 8.3.10 | Intracoronary fibrinolysis | Categorical | Was intracoronary fibrinolysis performed during PCI? | Yes - No |
| 8.4 | Revascularization surgery | Categorical | Was revascularization surgery performed as a main STEMI therapy? | Yes - No |
| 8.4.1 | Reason for surgery | Categorical | Reason for deciding on surgery | PCI frustrates Cardiogenic shock Mechanical complication  High-risk anatomy for PCI |
| 9 | **Reason for non-reperfusion** | Categorical | If NO to question 8.  Reason why the patient did not undergo any reperfusion therapy. | Contraindication to fibrinolysis/PCI  Lack of fibrinolytics /PCI Patient refusal  Late presentation 12 to 24 hours Late presentation 24 to 72 hours  Late presentation > 72 hours  Other. |
| 10 | LVEF after infarction | Quantitative | Evaluated by echocardiography during hospitalization (consider its lower value) | In percentage |
| 11 | Other medication or procedures during hospitalization |  |  |  |
| 11.1 | Inotropics/inodilators | Categorical | Did the patient receive dobutamine, dopamine or levosimendan during hospitalization? | Yes – No |
| 11.2 | Nitrates | Categorical | Did the patient receive nitrates during hospitalization? | Yes – No |
| 11.3 | Vasopressors | Categorical | Did the patient receive noradrenaline, vasopressin during hospitalization? | Yes – No |
| 11.4 | IAB | Categorical | Did the patient receive intra-aortic counterpulsation balloon during hospitalization? | Yes – No |
| 11.5 | NIV | Categorical | Did the patient require noninvasive ventilation? | Yes – No |
| 11.6 | Invasive MV | Categorical | Did the patient require invasive mechanical ventilation (ETT)? | Yes – No |
| 12 | Discharge medication |  |  |  |
| 12.1 | Aspirin | Categorical | Did the patient receive aspirin at discharge? | Yes – No |
| 12.2 | Clopidogrel | Categorical | Did the patient receive clopidogrel at discharge? | Yes – No |
| 12.3 | Prasugrel | Categorical | Did the patient receive prasugrel at discharge? | Yes – No |
| 12.4 | Ticagrelor | Categorical | Did the patient receive ticagrelor at discharge? | Yes – No |
| 12.5 | Nitrates | Categorical | Did the patient receive nitrates by patch or orally at discharge? | Yes – No |
| 12.6 | Betablocker | Categorical | Did the patient receive beta-blockers at discharge? | Yes – No |
| 12.7 | ACEI / ARA II | Categorical | Did the patient receive ACE inhibitors or ARA II at discharge? | Yes – No |
| 12.8 | Statins | Categorical | Did the patient receive statins at discharge? | Yes – No |
| 12.9 | Diuretics | Categorical | Did the patient receive diuretics at discharge? | Yes – No |
| 12.10 | Spironolactone | Categorical | Did the patient receive spironolactone at discharge? | Yes – No |
| 12.11 | Oral anticoagulants | Categorical | Did the patient receive oral anticoagulants at discharge? | Yes - No |
| 13 | **In-hospital events** | Categorical | Adverse events during hospitalization | Yes - No |
| 13.1 | Days of hospitalization | Quantitative | Number of days from the patient's arrival at the last hospital until discharge. | In days completed |
| 13.2 | Total in-hospital mortality | Categorical | Did the patient die during hospitalization? | Yes - No |
| 13.2.1 | In-hospital Cardiovascular Death | Categorical | Cardiac death during hospitalization | Yes - No |
| 13.2.2 | In-hospital sudden (arrhythmic) death | Categorical | Death due to arrhythmic cause (VT/VF) during hospitalization | Yes - No |
| 13.3 | Re-infarction | Categorical | New troponin elevation with or without ECG changes with or without symptoms during hospitalization | Yes - No |
| 13.4 | Post infarction VSD | Categorical | Presence of rupture of the interventricular septum due to infarction. | Yes - No |
| 13.5 | Papillary muscle rupture | Categorical | Rupture of the papillary muscle leading to acute mitral insufficiency | Yes - No |
| 13.6 | Free wall breakage | Categorical | Ventricular free wall rupture leading to contained pericardial effusion or frank cardiac tamponade | Yes - No |
| 13.6 | Cardiogenic shock | Categorical | Defined as blood pressure < 90 mmHg for more than 30 minutes associated with low cardiac index, signs of low cardiac output and need for inotropics and/or vasopressors | Yes - No |
| 13.7 | Symptomatic heart failure after infarction | Categorical | Presence of signs and/or symptoms of post-infarction heart failure (pulmonary crackles, jugular ingurgitation, third and/or fourth heart sounds, coldness of limbs, oliguria, slow capillary refill) | Yes - No |
| 13.8.1 | Ischemic stroke | Categorical | Presence of TIA, cerebral infarction during evolution in hospitalization | Yes - No |
| 12.8.2 | Hemorrhagic stroke | Categorical | Presence of cerebral hemorrhage in any of its presentations during hospitalization | Yes - No |
| 13.9 | High-grade AVB | Categorical | Presence of atrioventricular block of II degree Mobitz 2 or III degree at admission or during hospitalization | Yes - No |
| 13.10 | Major bleeding | Categorical | Presence of bleeding during hospitalization, assessed by hemoglobin drop > 5 or intra-cerebral localization | Yes - No |
| 14 | **30-day follow-up events** | Categorical | Adverse events at 30 days of follow up | Yes - No |
| 14.1 | Mortality at 30 days | Categorical |  | Yes - No |
| 14.1.1 | Cardiac death at 30 days | Categorical | Post-discharge cardiac death up to 30 days after discharge | Yes - No |
| 14.1.2 | Non-cardiac death at 30 days | Categorical | Post-discharge non-cardiac death up to 30 days post-discharge | Yes - No |
| 14.2 | Re-infarction | Categorical | New troponin elevation with or without ECG changes with or without symptoms post discharge up to 30 days | Yes - No |
| 14.3 | Re-hospitalization for heart failure | Categorical | Was the patient re-hospitalized after discharge for heart failure? | Yes - No |
| 15 | **Events per year** | Categorical | Adverse events after one year of follow up | Yes - No |
| 15.1 | Cardiovascular death | Categorical | Death from cardiac causes up to the control year | Yes - No |
| 15.2 | Stroke | Categorical | Ischemic/hemorrhagic stroke episode from 30 days to one year of follow-up | Yes - No |
| 15.3 | Hospitalization for Heart Failure | Categorical | Hospitalization for decompensated heart failure from 30 days to year of follow-up | Yes - No |

PCI: percutaneous coronary intervention, ECG: electrocardiogram, KK: Killip-Kimball classification, tPA: tissue plasminogen activator, TNK: tenecteplase, ARI: artery responsible for infarct, aVL: augmented vector left, aVF: augmented vector foot, STE: ST-segment elevation, TIMI: thrombolysis in myocardial infarction, PTCA: percutaneous transluminal coronary angioplasty, CABG: coronary artery bypass graft, GP: glycoprotein, STEMI: ST-segment elevation myocardial infarction, LVEF: left ventricular ejection fraction, IAB: intra-aortic counterpulsation balloon, NIV: noninvasive ventilation, MV: mechanical ventilation, ETT: endotracheal tube, ACEI: angiotensin-converting enzyme inhibitor, ARA II: angiotensin receptor antagonists, VT: ventricular tachycardia, VF: ventricular fibrillation, VSD: ventricular septal defect, TIA: transient ischemic attack, AVB: Atrioventricular block.
